# Association of dietary diversity of 6–23 months aged children with prenatal and postnatal obstetric care: evidence from a nationwide cross-sectional study

**DOI:** 10.1186/s41043-023-00470-7

**Published:** 2023-11-06

**Authors:** Khandaker Tanveer Ahmed, Md. Karimuzzaman, Guliva Nazneen Pinky, Dibbya Pravas Dasgupta, Labiba Rahman, Md Moyazzem Hossain, Azizur Rahman

**Affiliations:** 1 Department of Statistics, Jahangirnagar University, Savar, Dhaka, 1342 Bangladesh; 2 DREXEL Dornsife School of Public Health, DREXEL University, Philadelphia, USA; 3 Department of Public Health and Informatics, Jahangirnagar University, Savar, Dhaka, 1342 Bangladesh; 4 Center for Health Services and Policy Research, School of Population and Public Health, University of British Columbia, Vancouver, Canada; 5 School of Mathematics, Statistics, and Physics, Newcastle University, Newcastle Upon Tyne, NE1 7RU UK; 6 School of Computing, Mathematics and Engineering, Charles Sturt University, Wagga Wagga, NSW 2678 Australia

**Keywords:** Dietary diversity, Nutrition, Maternal and child health, Obstetric care

## Abstract

**Background:**

Dietary diversity is a key determinant of infant and young child eating patterns for a variety of food groups taken by children between the ages of 6–23 months. The study aimed to examine the association between prenatal and postnatal obstetric care factors of mother and child’s dietary diversity, and specific food practices in Bangladesh.

**Methods:**

This study analyzed the data of 2497 children between the age of 6–23 extracted from the latest countrywide Bangladesh Demographic Health Survey 2017–2018 and explored relationships between prenatal and postnatal obstetric care received by mother and dietary diversity score (DDS), minimum dietary diversity (MDD), and introduction of solid, semi-solid, and soft foods (ISSSF) of their children.

**Results:**

Findings revealed that ≥ 4 antenatal care (ANC) visits care visits increased the DDS (adjusted $\beta$: 0.32, 95% CI [0.21, 0.43]), increased the likelihood of MDD (AOR 1.54, 95% CI [1.23, 1.93]), and ISSSF (AOR 1.24, 95% CI [1.08, 1.48]), consuming eggs (AOR 1.47, 95% CI [1.23, 1.76]), and vitamin A vegetables and fruits (AOR 1.38, 95% CI [1.15, 1.66]). Moreover, DDS (adjusted *β*: 0.05, 95% CI [0.00, 0.11]) and MDD (AOR 1.66, 95% CI [1.31, 2.11]) are linked to childbirth in a medical facility. The C-section delivery influences the DDS (adjusted $\beta$: 0.05, 95% CI [0.00, 0.10]), MDD (AOR 1.39, 95% CI [1.10, 1.75]), and ISSSF (AOR 1.22, 95% CI [1.02, 1.48]). Besides, postnatal visits within 48 h of delivery linked to MDD (AOR 0.66, 95% CI [0.49, 0.89]) and ISSSF (AOR 0.76, 95% CI [0.59, 0.97]), and physicians or professionals providing postnatal checkups were significantly associated with DDS (adjusted $\beta$: 0.09, 95% CI [0.02, 0.16]), MDD (AOR 1.69, 95% CI [1.26, 2.26]), and ISSSF (AOR 1.30, 95% CI [1.04, 1.62]).

**Conclusion:**

Knowledge of child nutritional feeding should emphasize during prenatal and postnatal obstetric care of mother, particularly during antenatal and postnatal visits, C-section delivery, and birth in a healthcare facility to eradicate malnutrition and establish healthy child feeding practices.

**Supplementary Information:**

The online version contains supplementary material available at 10.1186/s41043-023-00470-7.

## Background

Dietary diversity is a crucial determinant of infant and young child feeding practices for a variety of food categories taken by children between the ages of 6 and 23 months [1]. Malnutrition is associated with both the quality and quantity of food consumed. There are 156 million children aged under-5 years who are suffering from stunting and other forms of malnutrition in low-income countries [2]. Children are suffering from different form of malnutrition in several countries [3–8]. Child malnutrition is more prevalent in lower-income countries due to the lack of affordable nutritious food options [9]. Micronutrient deficiency in pregnant women is explained mainly by inadequate food consumption and a lack of variety in Bangladesh. Compared to other developing nations, Bangladesh has a higher likelihood of female and child undernutrition and lower consumption of various food categories [10, 11]. As a consequence, tackling the simultaneous burden of stunting and malnutrition has proved to be one of the most challenging aspects of achieving Sustainable Development Goals. New guidelines of the World Health Organization (WHO) on prenatal care for getting particular micronutrient supplements as well as a balanced calorie and protein dietary supplementation were published recently [12]. According to the recommendations by WHO [32], complementary feeding with fewer than 5 food groups and a delayed introduction of dietary variety, particularly meals including animal protein, are the two main barriers to receiving nutritional diversity. Children aged 6–23 months should eat items from ≥ 5 of the 8 food categories, according to the dietary diversity indicator set by WHO [13]. The eight food groups including, staples, animal-source protein (meat/poultry/fish), fruits/vegetables containing vitamin A, other fruits and vegetables, eggs, milk and milk products, legumes, and breastfeeding. It is recommended to begin introducing dietary diversity at age of six months because only breastmilk will no longer fulfill the kid's nutritional needs. Vegetables, fruits, pureed, mashed, or semi-solid foods made from cereals, other protein-rich foods, and meat can be given to infants [1].

The “standard maternal, neonatal and child health (MNCH)” program in Bangladesh includes a complete maternal nutrition package to address maternal undernutrition, including free calcium supplements, monitoring weight growth during pregnancy, increasing dietary intake and diversity, and enough rest [14]. In recent years, the infant and young child-feeding (IYCF) practices among children between the ages of 6 and 23 months have been increased as a result of economic progress, the implementation of Comprehensive Emergency Obstetric and Newborn Care (CEmONC), and the introduction of telemedicine services, which operate a nationwide call center called Shashthya Batayon to provide health services (23% in 2014 to 34% in 2017). Despite this, more than 28% of children aged under-5 years are still stunted in 2019. Obesity rates among children under five years old have risen (from 1.6% in 2014 to 2.4%in 2019), whereas the prevalence of malnutrition has reduced (from 14.3% in 2014 to a mere 9.8% in 2019) [15, 16]. As a result, identifying additional risk factors for diet-related malnutrition has been critical for malnutrition research and solutions, as improving child nutrition is a national priority and a critical strategy for long-term economic development. Research has shown that, due to a shortage of appropriately trained health human resources, such as physicians, nurses, and midwives, there is a gap between principle and practice in public health facilities, jeopardizing the general public's accessibility [17]. In Bangladesh, pregnant women's intake of various micronutrients has declined as a result of the financial crisis [18]. Another finding is that more dietary variety is linked with improved nutrition among children living in developing nations [19]. There has been very little research examining the connection between prenatal and postnatal maternal obstetrical care and dietary variety in children from Bangladesh. According to one research, the minimal level of nutritional variety was 23.8% and rose to 28.8% in 2014. In children aged 18–23 months, the MDD was 32.5% in 2011; it rose to 42.8% in 2014 [20]. Between the ages of 6 and 23 months, 40% of Indonesian children do not meet the MDD criteria (consuming 4 or more food groups out of 7) [2]. In Asia, the minimum level of dietary variety in India, Nepal, and Sri Lanka was 15%, 34%, and 71% respectively [21, 22]. Bangladesh, in particular, has a low rate of nutritional variety, accounting for 41.9% of the population. The MDD falls to 19.8% in children aged 6 to 11 months, it nearly triples in 18 to 23 months aged children, reaching 59.7% [23, 24].

Additionally, women who lacked knowledge were more probable to postpone the starting of supplementary foods and therefore fail to fulfill MDD criteria [20]. Additionally, a similar study indicates that mothers with a greater degree of education are associated with more suitable supplementary feeding habits [21, 25]. Children whose mothers ingested more than five food categories (on a scale of 0–9) had 5–9 times more likelihood to reach MDD than children whose moms consumed three food groups [26]. Women's empowerment and access to information (through television, radio, and newspapers) are also related to better newborn and child nutrition outcomes, while a lack of media exposure (via television, radio, and newspapers) has been linked with an inadequate dietary variety [20, 27]. The association of prenatal and postnatal obstetric care, dietary diversity practices, and other related foods practices has not been investigated in the Bangladesh context, to the best of our knowledge. Hence, this study aimed to investigate the association between maternal prenatal and postnatal obstetrical care and the dietary diversity score (DDS), minimum dietary diversity (MDD), and introduction of solid, semi-solid, and soft foods (ISSSF) among children. This study may provide critical evidence for reconsidering and implementing policies addressing the beginning of nutritional knowledge practice in prenatal and postnatal obstetric care and the critical nature of prenatal care for improving nutritional practice in under-two-year-old children.

## Materials and methods

### Study setting and design

The study used secondary data from the 2017–18 BDHS, the eighth of its kind in the country, which began in 1993 and is Bangladesh's longest-running healthcare survey series. The analysis dataset included only data for ever-married women aged 15 to 49 years who had a child aged 6–23 months. The BDHS sample for 2017–18 is nationally representative and includes all non-institutional housing units. As a sample frame, the study used the “Bangladesh Bureau of Statistics (BBS)” list of enumeration areas (EAs) from the “2011 Population and Housing Census of the People's Republic of Bangladesh” [28]. Specifically, the association EA, which contains 120 homes, is the primary sample unit (PSU) for this study. Households were sampled in two phases throughout the study. Firstly, 675 EAs (urban: 250, rural: 425) were selected within the first opening, with the probability of selection increasing in direct proportion to the size of the EA. According to the DHS team's instructions, BBS took the sample in the first step. All of the selected EAs took part in a whole-house listing process to create a sample frame for the second step of household selection, which was conducted after the first stage. During the second round of sampling, a scientific sample of thirty families per EA was chosen from each division of the country.

Six different kinds of questionnaires were used in the 2017–18 BDHS: the Household Questionnaire, the Woman's Questionnaire, the Biomarker Questionnaire, two verbal autopsy questionnaires to gather information on causes of death among children under the age of 5, the Community Questionnaire, and the Fieldworker Questionnaire. First three questionnaires were derived from the sample forms created for the DHS-7 Program and had been modified for Bangladesh's context and needs while also considering the questions asked in earlier BDHS surveys. In this study, the Woman's Questionnaire was used. Women aged 15 to 49 who had ever been married provided information for the Woman's Questionnaire. The following questions elicited responses from women: Background information (for example, age, education, religion, and media exposure), reproductive history, family planning methods used and their source, antenatal, postpartum, and infant care, and breastfeeding, vaccinations for children, sickness and infant feeding techniques, getting married and activities sexual, preferences for fertility, background traits of husbands and women's employment. This research evaluated the data from the Woman's Questionnaire, where the respondents had a children aged six to twenty-three months. By searching as per the above-mentioned condition, a weighted sample of 2497 children were extracted and used in the study analysis. The details of the sampling procedure and methods of the weighted sample (mathematically adjusted) are available on the report of the Bangladesh Demographic and Health Survey-2017–18 [29].

### Independent variables

Obstetrical care covers labor, delivery, and the postpartum period as well as the treatment of both straightforward and complicated pregnancies. The treatment of expecting mothers, the health of the growing child, labor and delivery, and postpartum care are the main areas of focus in the medical specialty of obstetrics. Obstetric care is crucial to ensuring a simple labor and delivery and, in the event that intervention is necessary, that care be given promptly and securely. In this study, the covariates related to prenatal and postnatal obstetric care were considered, such as, the prenatal care attributes included the number of antenatal visits before child delivery (4 or more and fewer than 4) and the location of child delivery (at home or with health facility). The postnatal care attributes included the method of delivery (vaginal or C-section), the time interval between postnatal visits (48 h or more than 48 h after delivery), and the postnatal check-up provider (physician/professional). These variables were used as independent variables to assess the association with child feeding practices. A comparable previous research study performed in Indonesia, Tanzania, and five South Asian nations provided the basis for selecting the independent variables [2, 30, 31]. These studies examined all of these selected predictor variables separately in their respective studies of a similar nature. According to the WHO standard level, 4 or more antenatal visits were required, and the standard timing of postnatal visits was also a dichotomous variable of within 48 h of delivery or later than that, based on WHO (2015) guidelines [32].

### Dependent variables

The DDS was computed using cereals, tubers, and roots; dairy products (milk, cheese, and yogurt); legumes and nuts; eggs; flesh (fish, meat, poultry, and liver/organ meats); vitamin A-rich fruits and vegetables; and other fruits and vegetables, and total number of food groups consumed out of the 7 mentioned was the score of the DDS. The MDD is the proportion of children aged 6 to 23 months who consumed food from five or more categories in the preceding 24 h from a suggested 8 food groups by WHO [13], answered to a list-based method where questions about all the food categories were asked. If a child had received at least 5 foods out of the 8 defined foods, the MDD was achieved. The percentage of child who received solid, semi-solid, or soft foods was determined to be the ISSSF rate for the age group of 6–8 months. ISSSF was previously used as indication of child’s complementary dietary diversity in similar type of studies [30]. Following previous research [2, 26, 33], many covariates were included as adjustments in the analysis, including maternal age (15–24, 25–34, and 35–49 years of age interval), maternal education (not educated, primary, secondary, or higher), maternal occupation (not working, services, agriculture, and household/domestic work), and wealth index (poorest, poorer, middle, richer and richest). Wealth index categories along with combined wealth score well defined and structured in the BDHS 2017–18 dataset for the respondents.

### Statistical analysis

The data analysis included children aged 6–23 months, resulting in a total of 2497 children. The analytics included information on the number of children, the timing of postnatal visits, and MDD. Frequencies and weighted percentages were used to represent all independent and dependent variables. We used unadjusted univariate Poisson regression to examine the relationships between independent factors and children's DDS, as DDS is a score of the count of the total number of food groups consumed out of the 8 mentioned food groups. The goodness of fit of the Poisson models indicated that the models were well fitted for the data, with deviance goodness of fit and Pearson goodness of fit suggesting the data follows Poisson distribution as all the p-values for the models’ were close to 1. Additionally, unadjusted logistic regression was carried out to explore the relationships between different variables and MDD, as well as the ISSSF and their intake in children. To address potential confounding effects, the analysis accounted for maternal care practices during pregnancy when assessing the association between child feeding practices and intra- and postnatal care practices. Adjustments were made by including maternal care practices as covariates in the regression models. To account for potential confounding effects, the analysis included adjustments for predictor variables such as maternal age, occupation, and wealth index. Poisson and logistic regression models were utilized, and health facility delivery under place of delivery was used as an adjustment specifically for C-section delivery. This adjustment aimed to minimize any confounding effects that may arise from the mode of delivery, ensuring a more accurate assessment of the association between the predictor variables and the outcomes.

To check multicollinearity among the predictor variables with adjusting covariates, the variance inflation factor (VIF) was used. Mean VIF among the predictor variables, the number of antenatal visits before child delivery (4 or more and fewer than 4), the location of child delivery (at home or with a health facility), the method of delivery (vaginal or C-section), the time interval between postnatal visits (48 h or more than 48 h after delivery), and the postnatal check-up provider (physician/professional), and the adjusting covariates were 1.12, 1.15, 1.12, 1.14 and 1.13 respectively, which indicates no concerning existence of multicollinearity. In the adjusted regression analysis, determinants of interest were defined as independent variables that were statistically significant at the 5% level. Moreover, the authors investigated the effect of selected independent factors on the DDS, MDD, and ISSSF in urban and rural settings. The coefficient plots depicted the adjusted correlations between the intake of seven food categories and the target variables. All analyzes take into consideration the survey's sample methodology and weighting. For statistical analysis, Stata version 16 was utilized.

## Results

### Socio-economic characteristics and maternal care information of mothers in time of pregnancy and delivery

More than half of the children (51.54%) aged of 6 to 23 months were male, and most of them were from rural areas (66.48%). The average age of the children was 14.43 (± 5.18) months. Mothers of the majority of children were between the ages of 15 and 24 (53.82%) with average age of 25.80 (± 5.65) years, unemployed (61.23%), and educated at least to the secondary level (48.02%). The majority of mothers (52.7%) had fewer than 4 prenatal visits, gave birth in healthcare facilities (50.98%), and delivered vaginally (65.84%). Mothers had information on when to schedule a postnatal visit following delivery; the majority of them had scheduled a postnatal visit within 48 h (73.4%) and, mothers who had information on postnatal check-up providers, the majority had used physicians and other professionals (53.18%). Tables 1 and 2 contain all of the frequency analyzes of mothers' socioeconomic characteristics and maternal care during pregnancy, as well as the characteristics of their delivery.

**Table 1:** Socio-economic characteristics of 6–23 months old children and their caregivers

Characteristics	Frequency (*n* = 2497)	Weighted %
*Age of child (in months)*		
Mean (± standard deviation)	14.43 (± 5.18)	
*Maternal Age (in years)*		
Mean (± standard deviation)	25.80 (± 5.65)	
*Sex of child*		
Male	1287	51.54
Female	1210	48.46
*Residence*		
Urban	837	33.52
Rural	1660	66.48
*Age category (years)*		
15–24	1344	53.82
25–34	993	39.77
35–49	160	6.41
*Maternal occupation*		
Not working	1529	61.23
Services	274	10.97
Agriculture	680	27.23
Household and domestic	14	0.56
*Maternal education*		
Not educated	151	6.05
Primary	692	27.71
Secondary	1199	48.02
Higher	455	18.22
*Wealth index*		
Poorest	533	21.35
Poorer	526	21.07
Middle	437	17.50
Richer	515	20.62
Richest	486	19.46

**Table 2:** Maternal care in time of pregnancy and delivery

Characteristics	Frequency	Weighted %
*Number of antenatal visits*	*2497*	
0–3	1316	52.70
≥ 4	1181	47.30
*Place of delivery*	*2497*	
At home	1224	49.02
With health facility	1273	50.98
*Delivery method*	*2497*	
Vaginal	1644	65.84
C-section	853	34.16
*Timing of postnatal visit after delivery*	*1654*	
After 48 h	440	26.60
Within 48 h	1214	73.40
*Postnatal checkup provider*	*1653*	
Unprofessional personnel	774	46.82
Physician or other professionals	879	53.18

According to the information on children's food consumption in the preceding 24 h, 91.47% were being breastfed, 86.18% consumed grains, 41.87% consumed eggs, 38.69% consumed vitamin A-containing foods, 28.38% consumed other fruits and vegetables, and 23.97% consumed lentils and nuts. Only 7.05% of children consumed meat of any kind, and 2.15% consumed dairy products in the preceding 24 h. The mean score for dietary diversity was 2.25 (± 1.32) with the range of the mean dietary diversity score being 0–6, and medians of dietary diversity score at 25th, 50th, and 75th percentiles were respectively 1, 2, and 3. Only 16.74% of children achieved MDD. Moreover, the majority of children (61.81%) consumed solid, semi-solid, and soft foods.

### Maternal care related determinants of DDS, MDD, and ISSSF

A significant positive impact was found between ≥ 4 antenatal care (ANC) visits with higher DDS, while also having higher odds with MDD and ISSSF in comparison with less than 4 visits (Table 3 for DDS, Table 4 for MDD, and Table 5 for ISSSF). Also, 4 or more ANC visits provided 1.47 times higher likelihood of consuming eggs.

**Table 3:** Association between maternal care parameters of mothers in time of pregnancy and delivery with DDS

Characteristics	Dietary diversity score					
	Crude			Adjusted		
	$\beta$	95% CI	$p$-value	$\beta$	95% CI	$p$-value
*Number of antenatal visits*						
0–3	Ref					
≥ 4	0.19	0.14, 0.24	< 0.001	0.14	0.09, 0.20	< 0.001
*Place of delivery*						
At home	Ref					
With health facility	0.12	0.07, 0.17	< 0.001	0.05	0.00, 0.11	0.042
*Delivery method*						
Vaginal	Ref					
C-section	0.11	0.06, 0.17	< 0.001	0.05	0.00, 0.10	0.041
*Postnatal visit after delivery*						
After 48 h	Ref					
Within 48 h	− 0.14	− 0.21, − 0.07	< 0.001	− 0.06	− 0.13, 0.02	0.136
*Postnatal checkup provider*						
Unprofessional personnel	Ref					
Physicians/ professionals	0.16	0.10, 0.22	< 0.001	0.09	0.02, 0.16	0.008

*Ref*. Reference category, Adjusted by maternal age, occupation and wealth index

**Table 4:** Association between maternal care parameters of mothers in time of pregnancy and delivery with MDD

Characteristics	Minimum dietary diversity					
	Crude			Adjusted		
	OR	95% CI	*p*-value	OR	95% CI	*p*-value
*Number of antenatal visits*						
0–3	Ref					
≥ 4	1.95	1.57, 2.41	< 0.001	1.54	1.23, 1.93	< 0.001
*Place of delivery*						
At home	Ref					
With health facility	2.11	1.69, 2.63	< 0.001	1.66	1.31, 2.11	< 0.001
*Delivery method*						
Vaginal	Ref					
C-section	1.80	1.45, 2.23	< 0.001	1.39	1.10, 1.75	0.005
*Postnatal visit after delivery*						
After 48 h	Ref					
Within 48 h	0.48	0.37, 0.62	< 0.001	0.66	0.49, 0.89	0.007
*Postnatal checkup provider*						
Unprofessional personnel	Ref					
Physicians/ professionals	2.19	1.67, 2.88	< 0.001	1.69	1.26, 2.26	< 0.001

*Ref*. Reference category, Adjusted by maternal age, occupation and wealth index

**Table 5:** Association between maternal care parameters of mothers in time of pregnancy and delivery with ISSSF

Characteristics	Introduction of solid, semi-solid and soft foods					
	Crude			Adjusted		
	OR	95% CI	*p*-value	OR	95% CI	*p*-value
*Number of antenatal visits*						
0–3	Ref					
≥ 4	1.24	1.05, 1.46	0.011	1.24	1.04, 1.48	0.016
*Place of delivery*						
At home	Ref					
With health facility	1.09	0.92, 1.28	0.304	1.12	0.93, 1.34	0.203
*Delivery method*						
Vaginal	Ref					
C-section	1.18	0.99, 1.40	0.066	1.22	1.02, 1.48	0.034
*Postnatal visit after delivery*						
After 48 h	Ref					
Within 48 h	0.79	0.63, 1.00	0.052	0.76	0.59, 0.97	0.030
*Postnatal checkup provider*						
Unprofessional personnel	Ref					
Physicians/ professionals	1.25	1.02, 1.53	0.032	1.30	1.04, 1.62	0.019

*Ref*. Reference category, Adjusted by maternal age, occupation and wealth index

Delivery at health care facilities had shown a significant positive impact with the increase of DDS and provided 1.66 times more likelihood of MDD compared to children delivered at home, but the delivery place was not significantly linked with ISSS and food consumption. In the case of the delivery method, DDS was associated significantly with C-section delivery with higher odds, 1.39 times higher odds of MDD, and higher odds by 1.22 times for ISSSF when compared to vaginal delivery. On the other hand, time to postnatal visits after delivery was not associated with DDS, as well as food consumption for children, but for MDD and ISSSF, postnatal visits within 48 h showed significantly lower odds in both cases. Physicians or professionals providing postnatal checkups were significantly associated with DDS, with 1.69 times higher odds for MDD and 1.30 times higher odds for ISSSF (Table 3 for DDS, Table 4 for MDD, and Table 5 for ISSSF).

Mothers residing in urban areas and attending four or more antenatal care visits exhibited significantly higher DDS, MDD, and ISSSF compared to those with less than four antenatal care visits. C-section delivery was only substantially linked with ISSSF, with odds ratios of 1.39 times greater. On the other hand, postnatal checkups administered by physicians or professionals to mothers of 6–23-month-old children living in urban areas were associated with significantly higher DDS and 2.31-fold higher odds of MDD in comparison to unprofessional personnel, but the association with ISSSF and ISSSF was not significant (adjusted odds ratio in Additional file 1: Table S1 for DDS, Additional file 1: Table S2 for MDD, and Additional file 1: Table S3 for ISSSF).

Mothers living in rural areas who received ≥ 4 antenatal care was significantly related with higher DDS, showed 1.56 times higher likelihood of MDD compared to less than 4 visits, although no association with ISSSF was found. Delivery at a health care facility in rural areas had shown significant association only with MDD, providing 1.82 times more likelihood of MDD than delivery at home. Meanwhile, no significant relationship was found for DDS, food consumption of different types, and ISSSF with the delivery place in rural areas (adjusted odds ratio in Additional file 1: Table S4 for DDS, Additional file 1: Table S5 for MDD, and Additional file 1: Table S6 for ISSSF).

In contrast, delivery by C-section and postnatal visits within 48 h of delivery, both for the mothers in rural areas, had a significant association with only MDD, not with DDS and ISSSF. Delivery by C-section had 1.48 times higher likelihood of MDD for mothers in rural areas compared to vaginal delivery. However, postnatal visits within 48 h of delivery in rural areas provided lower likelihood by 0.55 times in comparison with delayed postnatal visits and visiting postnatal care after 48 h of delivery, for MDD. Moreover, postnatal checkup given by physicians or professionals to mothers who lived in rural areas was not associated with DDS and food consumption of various groups. However, it was associated significantly with MDD and ISSSF, providing 1.43 times higher odds for MDD and 1.30 times higher odds for ISSSF compared to postnatal checkup by any unprofessional personnel (adjusted odds ratio in Additional file 1: Table S4 for DDS, Additional file 1: Table S5 for MDD, and Additional file 1: Table S6 for ISSSF).

## Discussion

Using data from the BDHS (2017–18), this study examined the association between maternal care parameters with DDS, MDD, and ISSSF. The findings indicate a positive relationship between DDS, MDD, and the ISSSF during 4 or more prenatal care visits. Additionally, having 4 or more prenatal visits increased the likelihood of consuming eggs and fruits, and vegetables high in vitamin A. At the same time, no correlation was found between ISSSF and different food consumption patterns in mothers living in rural areas who received 4 or more prenatal visits. This could be because rural women continue to lack access to counseling regarding the ISSSF and different food categories' consumption patterns. According to a 2018 study in Bangladesh, mothers who lived in rural areas received on average less than three ANC visits, and only 6% received the recommended at least 8 ANC visits, which may explain why mothers in remote regions continue to be unable to receive proper counseling [34].

In comparison to children delivered at home, birth in a health care facility was linked with increased rates of DD and MDD but not with the ISSSF or food consumption. According to an Indonesian study, having a properly educated delivery attendant and giving birth in a health institution were connected with a higher incidence of dietary diversity among children [2]. Previous researchers highlighted that there is a good correlation between giving birth in a health facility as well as having a professionally trained delivery attendant with a more variety diet for children [23, 35–37]. Contrary to our pooled sample, delivery in an urban or rural health care facility was not linked with DD or ISSSF. MDD followed the same pattern in urban regions as it did in rural areas but was significantly linked with delivery in a health care facility.

Furthermore, a higher DDS, greater MDD, and higher ISSSF were found to be associated with C-section delivery. However, upon conducting a stratified analysis, significant differences were observed. MDD was associated with C-section delivery only in rural mothers, while ISSSF was associated with MDD exclusively in urban mothers. The alarming increase in c-section deliveries in Bangladesh explains the discrepancies, as it means that people are receiving c-sections even when they are not necessary, invalidating the data. Over 60% of C-sections in Bangladesh were carried out in for-profit private facilities [38]. Improved socioeconomic status, increased education, lower birth order, increased age, increased use of Antenatal Care, and an adverse prenatal and postnatal obstetric history all increased the likelihood of a C-section delivery [38, 39].

The time between delivery and the first postnatal visit was not associated with DDS or food consumption in children, but the first postnatal visit within 48 h was linked with a lowered odd of MDD and ISSSF. Postnatal visits within 48 h of delivery in rural areas were associated with a lower likelihood of delayed postnatal visits and postnatal care after 48 h for MDD. This could be due to the fact that a significant proportion (52.8%) of postnatal visits in rural areas were made by untrained personnel, with 93.2% of these visits occurring within 48 h of delivery. On the other hand, among the visits made by professionals, 38.8% occurred after 48 h of delivery. Additionally, postnatal checkups by physicians or other professionals were associated with a significantly higher DDS, a higher probability of MDD, and a greater likelihood of ISSSF. Our stratified analysis by place of residence revealed similar findings for mothers of 6–23-month-old children who lived in urban areas, except that no association with ISSSF or consumption of different food groups by children with a 24-h recall was observed. On the other hand, postnatal checkups performed by physicians or professionals on mothers living in rural areas were only associated with MDD and the ISSSFF in a statistically significant manner. There could be various reasons for this, but prenatal visits are likely more effective than postnatal care at improving children's nutritional quality [2]. Delayed or fewer postnatal visits were linked to a reduced incidence of MDD, findings of another study revealed a negative correlation between postnatal visits and a minimally tolerable diet, probably due to substandard postnatal care quality [23, 31, 36, 40, 41].

Previous research has highlighted the importance of maternal care parameters, such as prenatal care visits and delivery in healthcare facilities, in influencing child feeding practices and dietary diversity [2]. Studies in Bangladesh and Indonesia have shown positive associations between adequate antenatal care visits and improved dietary diversity among children [2, 34]. Additionally, the role of postnatal checkups by physicians or professionals in enhancing child feeding practices has been documented [23, 35]. The current study, using data from the BDHS (2017–18), found that in Bangladesh, mothers who had four or more prenatal care visits had a higher likelihood of consuming eggs, fruits, and vegetables high in vitamin A. Birth in a healthcare facility was associated with increased rates of dietary diversity and minimum dietary diversity (MDD) among children. Furthermore, C-section delivery was linked to higher dietary diversity scores (DDS), MDD, and minimum meal frequency (ISSSF). The timing of the first postnatal visit within 48 h was associated with a reduced risk of MDD and ISSSF. The findings suggest that adequate prenatal care visits and delivery in healthcare facilities are associated with improved child feeding practices and dietary diversity. However, disparities were observed between rural and urban areas, indicating that access to counseling and education on infant and young child feeding practices remains limited in rural regions. The alarming rise in unnecessary C-section deliveries in Bangladesh highlights the need for better adherence to evidence-based practices and appropriate utilization of healthcare resources. The association between postnatal checkups and improved child feeding practices underscores the importance of postnatal care in ensuring optimal nutrition for children. Addressing the quality and timing of postnatal care can potentially enhance child feeding practices and contribute to better nutritional outcomes.

### Limitation and future scope

Because of the cross-sectional study, a causal relationship between prenatal and postnatal obstetrical care and child dietary diversity may not be discernible. Although previous research has established a link between maternal education and health concerns such as obesity [19], mother education was excluded from the adjusted regression analysis as it exhibited a strong positive correlation with the wealth index, but its inclusion did not significantly affect the regression results. Additionally, there may be some components that were not included in this study but have a significant link with the study variables due to their non-availability in the BDHS 2017–18 survey data set, such as who delivered the child, prenatal and postnatal obstetrical history, previous pregnancy complications for the mother, and baseline BMI for all family members of a household, that could have an effect in this study. This is the first study in Bangladesh to explore the relationship between prenatal and postnatal obstetric care factors and consumption of individual food groups and introduction to solid, semi-solid, and soft foods. The study’s findings have programmatic implications and will aid efforts to assess and improve the diversity and micronutrient sufficiency of mothers’ and children’s diets. Future studies should concentrate on maternal care and how it affects the consumption of food groups and the ISSSF in order to guide future policies and initiatives.

## Conclusion

As suggested in this study, prenatal and postnatal obstetrical care taken from any health facility and trained and professional physicians, nurses, or midwives was highly associated with dietary diversity and proper food consumption. So, proper guidance and counseling should be provided to the mothers and families around the country regarding health facilities given prenatal and postnatal obstetrical care and their importance, as it will undoubtedly help eradicate malnutrition and establish healthy child feeding practices. Maternal education should be collaborative with prenatal and postnatal obstetrical care-related information and must be provided to the community level in Bangladesh’s urban and rural areas. Community clinics and health workers must reach out to the underdeveloped part of the societies to spread the education related to prenatal and postnatal obstetric care, and mothers also must be elucidated in this regard in time of antenatal care visits and postnatal visits. It was found that ≥ 4 antenatal care (ANC) visits and postnatal care provided by professionals associates positively to dietary diversity of children, and so, administrative steps should be implemented rigidly to achieve the goal of providing more than four antenatal visits and professional postnatal care to every mother in both urban and rural areas. The findings emphasize the importance of addressing maternal pregnancy care factors, such as antenatal and postnatal visits, C-section delivery, and birth in a healthcare facility, in promoting child dietary diversity practices. Implementation of these suggested changes has the potential to improve children's dietary diversity and mitigate malnutrition.

### Supplementary Information

**Additional file 1**. Supplementary Tables.

## Data Availability

The secondary data, BDHS 2017–18, used in this study is available in the website of the DHS Program (http://dhsprogram.com/data/available-datasets.cfm).
