# Supplementary material for: Association of dietary diversity of 6–23 months aged children with prenatal and postnatal obstetric care: evidence from a nationwide cross-sectional study
Source: J Health Popul Nutr. 2023 Nov 6;42:120. doi: 10.1186/s41043-023-00470-7 (PMC10629119; doi:10.1186/s41043-023-00470-7)
Supplement: Supplementary file 1 — Additional file 1. Supplementary Tables. [file 41043_2023_470_MOESM1_ESM.docx]

**Association of Dietary Diversity of 6-23 Months Aged Children with Prenatal and Postnatal Obstetric Care: Evidence from a Nationwide Cross-sectional Study**

**Supplementary Table 1:** Association between maternal care parameters of mothers of living in urban areas in time of pregnancy and delivery with DDS

|  | **Dietary diversity score** | | | | | |
| --- | --- | --- | --- | --- | --- | --- |
|  | **Crude** | | | **Adjusted** | | |
|  | *β* | 95% CI | *p*-value | *β* | 95% CI | *p*-value |
| *Number of antenatal visits* |  |  |  |  |  |  |
| 0-3 antenatal visits | Ref. |  |  |  |  |  |
| ≥4 antenatal visits | 0.22 | 0.13, 0.31 | <0.001 | 0.17 | 0.07, 0.27 | 0.001 |
| *Place of delivery* |  |  |  |  |  |  |
| Home delivery | Ref. |  |  |  |  |  |
| Health facility delivery | 0.12 | 0.03, 0.21 | 0.012 | 0.04 | -0.06, 0.14 | 0.398 |
| *Delivery method* |  |  |  |  |  |  |
| Vaginal | Ref. |  |  |  |  |  |
| C-section | 0.14 | 0.05, 0.22 | 0.002 | 0.07 | -0.03, 0.16 | 0.170 |
| *Postnatal visit after delivery* |  |  |  |  |  |  |
| After 48 hours | Ref. |  |  |  |  |  |
| Within 48 hours | -0.11 | -0.22, 0.00 | 0.045 | -0.04 | -0.16, 0.08 | 0.516 |
| *Postnatal checkup provider* |  |  |  |  |  |  |
| Unprofessional personnel | Ref. |  |  |  |  |  |
| Physicians/ professionals | 0.22 | 0.11, 0.34 | <0.001 | 0.16 | 0.03, 0.29 | 0.016 |

**Note:** Adjusted by maternal age, occupation and wealth index

**Supplementary Table 2:** Association between maternal care parameters of mothers living in urban areas in time of pregnancy and delivery with MDD

|  | **Minimum dietary diversity** | | | | | |
| --- | --- | --- | --- | --- | --- | --- |
|  | **Crude** | | | **Adjusted** | | |
|  | OR | 95% CI | *p*-value | OR | 95% CI | *p*-value |
| *Number of antenatal visits* |  |  |  |  |  |  |
| 0-3 antenatal visits | Ref. |  |  |  |  |  |
| ≥4 antenatal visits | 1.83 | 1.28, 2.63 | 0.001 | 1.48 | 1.01, 2.17 | 0.046 |
| *Place of delivery* |  |  |  |  |  |  |
| Home delivery | Ref. |  |  |  |  |  |
| Health facility delivery | 1.74 | 1.20, 2.52 | 0.003 | 1.32 | 0.87, 1.98 | 0.170 |
| *Delivery method* |  |  |  |  |  |  |
| Vaginal | Ref. |  |  |  |  |  |
| C-section | 1.61 | 1.15, 2.25 | 0.005 | 1.23 | 0.85, 1.77 | 0.271 |
| *Postnatal visit after delivery* |  |  |  |  |  |  |
| After 48 hours | Ref. |  |  |  |  |  |
| Within 48 hours | 0.67 | 0.45, 1.01 | 0.056 | 0.89 | 0.57, 1.39 | 0.623 |
| *Postnatal checkup provider* |  |  |  |  |  |  |
| Unprofessional personnel | Ref. |  |  |  |  |  |
| Physicians/ professionals | 2.87 | 1.74, 4.75 | <0.001 | 2.31 | 1.34, 3.98 | 0.003 |

**Note:** Adjusted by maternal age, occupation and wealth index

**Supplementary Table 3:** Association between maternal care parameters of mothers living in urban areas in time of pregnancy and delivery with ISSSF

|  | **Introduction of solid, semi-solid and soft foods** | | | | | |
| --- | --- | --- | --- | --- | --- | --- |
|  | **Crude** | | | **Adjusted** | | |
|  | OR | 95% CI | *p*-value | OR | 95% CI | *p*-value |
| *Number of antenatal visits* |  |  |  |  |  |  |
| 0-3 antenatal visits | Ref. |  |  |  |  |  |
| ≥4 antenatal visits | 1.29 | 0.96, 1.73 | 0.096 | 1.38 | 1.01, 1.90 | 0.046 |
| *Place of delivery* |  |  |  |  |  |  |
| Home delivery | Ref. |  |  |  |  |  |
| Health facility delivery | 1.05 | 0.78, 1.41 | 0.759 | 1.15 | 0.83, 1.61 | 0.399 |
| *Delivery method* |  |  |  |  |  |  |
| Vaginal | Ref. |  |  |  |  |  |
| C-section | 1.25 | 0.93, 1.68 | 0.136 | 1.39 | 1.01, 1.92 | 0.045 |
| *Postnatal visit after delivery* |  |  |  |  |  |  |
| After 48 hours | Ref. |  |  |  |  |  |
| Within 48 hours | 0.82 | 0.56, 1.20 | 0.305 | 0.74 | 0.49, 1.11 | 0.148 |
| *Postnatal checkup provider* |  |  |  |  |  |  |
| Unprofessional personnel | Ref. |  |  |  |  |  |
| Physicians/ professionals | 1.09 | 0.74, 1.61 | 0.668 | 1.24 | 0.80, 1.91 | 0.331 |

**Note:** Adjusted by maternal age, occupation and wealth index

**Supplementary Table 4:** Association between maternal care parameters of mothers living in rural areas in time of pregnancy and delivery with DDS

|  | **Dietary diversity score** | | | | | |
| --- | --- | --- | --- | --- | --- | --- |
|  | **Crude** | | | **Adjusted** | | |
|  | *β* | 95% CI | *p*-value | *β* | 95% CI | *p*-value |
| *Number of antenatal visits* |  |  |  |  |  |  |
| 0-3 antenatal visits | Ref. |  |  |  |  |  |
| ≥4 antenatal visits | 0.16 | 0.09, 0.22 | <0.001 | 0.12 | 0.05, 0.19 | <0.001 |
| *Place of delivery* |  |  |  |  |  |  |
| Home delivery | Ref. |  |  |  |  |  |
| Health facility delivery | 0.10 | 0.03, 0.16 | 0.004 | 0.05 | -0.01, 0.12 | 0.133 |
| *Delivery method* |  |  |  |  |  |  |
| Vaginal | Ref. |  |  |  |  |  |
| C-section | 0.08 | 0.00, 0.15 | 0.029 | 0.03 | -0.04, 0.11 | 0.359 |
| *Postnatal visit after delivery* |  |  |  |  |  |  |
| After 48 hours | Ref. |  |  |  |  |  |
| Within 48 hours | -0.13 | -0.22, -0.03 | 0.008 | -0.06 | -0.16, 0.03 | 0.222 |
| *Postnatal checkup provider* |  |  |  |  |  |  |
| Unprofessional personnel | Ref. |  |  |  |  |  |
| Physicians/ professionals | 0.11 | 0.03, 0.19 | 0.008 | 0.06 | -0.01, 0.14 | 0.134 |

**Note:** Adjusted by maternal age, occupation and wealth index

**Supplementary Table 5:** Association between maternal care parameters of mothers living in rural areas in time of pregnancy and delivery with MDD

|  | **Minimum dietary diversity** | | | | | |
| --- | --- | --- | --- | --- | --- | --- |
|  | **Crude** | | | **Adjusted** | | |
|  | OR | 95% CI | *p*-value | OR | 95% CI | *p*-value |
| *Number of antenatal visits* |  |  |  |  |  |  |
| 0-3 antenatal visits | Ref. |  |  |  |  |  |
| ≥4 antenatal visits | 1.85 | 1.40, 2.43 | <0.001 | 1.56 | 1.17, 2.07 | 0.002 |
| *Place of delivery* |  |  |  |  |  |  |
| Home delivery | Ref. |  |  |  |  |  |
| Health facility delivery | 2.16 | 1.64, 2.86 | <0.001 | 1.82 | 1.36, 2.45 | <0.001 |
| *Delivery method* |  |  |  |  |  |  |
| Vaginal | Ref. |  |  |  |  |  |
| C-section | 1.78 | 1.34, 2.36 | <0.001 | 1.48 | 1.10, 1.99 | 0.010 |
| *Postnatal visit after delivery* |  |  |  |  |  |  |
| After 48 hours | Ref. |  |  |  |  |  |
| Within 48 hours | 0.43 | 0.30, 0.62 | <0.001 | 0.55 | 0.37, 0.82 | 0.003 |
| *Postnatal checkup provider* |  |  |  |  |  |  |
| Unprofessional personnel | Ref. |  |  |  |  |  |
| Physicians/ professionals | 1.73 | 1.24, 2.43 | 0.001 | 1.43 | 1.00, 2.04 | 0.048 |

**Note:** Adjusted by maternal age, occupation and wealth index

**Supplementary Table 6:** Association between maternal care parameters of mothers of 6-23 months old children living in rural areas in time of pregnancy and delivery with ISSSF

|  | **Introduction of solid, semi-solid and soft foods** | | | | | |
| --- | --- | --- | --- | --- | --- | --- |
|  | **Crude** | | | **Adjusted** | | |
|  | OR | 95% CI | *p*-value | OR | 95% CI | *p*-value |
| *Number of antenatal visits* |  |  |  |  |  |  |
| 0-3 antenatal visits | Ref. |  |  |  |  |  |
| ≥4 antenatal visits | 1.14 | 0.93, 1.40 | 0.208 | 1.15 | 0.93, 1.42 | 0.190 |
| *Place of delivery* |  |  |  |  |  |  |
| Home delivery | Ref. |  |  |  |  |  |
| Health facility delivery | 1.04 | 0.85, 1.27 | 0.709 | 1.09 | 0.88, 1.35 | 0.414 |
| *Delivery method* |  |  |  |  |  |  |
| Vaginal | Ref. |  |  |  |  |  |
| C-section | 1.07 | 0.86, 1.33 | 0.542 | 1.14 | 0.91, 1.44 | 0.257 |
| *Postnatal visit after delivery* |  |  |  |  |  |  |
| After 48 hours | Ref. |  |  |  |  |  |
| Within 48 hours | 0.86 | 0.63, 1.16 | 0.315 | 0.81 | 0.58, 1.11 | 0.196 |
| *Postnatal checkup provider* |  |  |  |  |  |  |
| Unprofessional personnel | Ref. |  |  |  |  |  |
| Physicians/ professionals | 1.24 | 0.97, 1.58 | 0.085 | 1.30 | 1.00, 1.68 | 0.047 |

**Note:** Adjusted by maternal age, occupation and wealth index

**Supplementary Table 7:** Association between maternal care parameters of mothers in time of pregnancy and delivery with DDS

| **Characteristics** | **Dietary diversity score** | | | | | |
| --- | --- | --- | --- | --- | --- | --- |
|  | **Crude** | | | **Adjusted** | | |
|  | *β* | 95% CI | *p*-value | *β* | 95% CI | *p*-value |
| *Number of antenatal visits* |  |  |  |  |  |  |
| 0-3 | Ref. |  |  |  |  |  |
| ≥4 | 0.19 | 0.14, 0.24 | <0.001 | 0.14 | 0.09, 0.20 | <0.001 |
| *Place of delivery* |  |  |  |  |  |  |
| At home | Ref. |  |  |  |  |  |
| With health facility | 0.12 | 0.07, 0.17 | <0.001 | 0.05 | 0.00, 0.11 | 0.042 |
| *Delivery method* |  |  |  |  |  |  |
| Vaginal | Ref. |  |  |  |  |  |
| C-section | 0.11 | 0.06, 0.17 | <0.001 | 0.05 | 0.00, 0.10 | 0.041 |
| *Postnatal visit after delivery* |  |  |  |  |  |  |
| After 48 hours | Ref. |  |  |  |  |  |
| Within 48 hours | -0.14 | -0.21, -0.07 | <0.001 | -0.06 | -0.13, 0.02 | 0.136 |
| *Postnatal checkup provider* |  |  |  |  |  |  |
| Unprofessional personnel | Ref. |  |  |  |  |  |
| Physicians/ professionals | 0.16 | 0.10, 0.22 | <0.001 | 0.09 | 0.02, 0.16 | 0.008 |

**Note:** Ref.: Reference category; Adjusted by maternal age, occupation and wealth index

**Supplementary Table 8:** Association between maternal care parameters of mothers in time of pregnancy and delivery with MDD

| **Characteristics** | **Minimum dietary diversity** | | | | | |
| --- | --- | --- | --- | --- | --- | --- |
|  | **Crude** | | | **Adjusted** | | |
|  | OR | 95% CI | *p*-value | OR | 95% CI | *p*-value |
| *Number of antenatal visits* |  |  |  |  |  |  |
| 0-3 | Ref. |  |  |  |  |  |
| ≥4 | 1.95 | 1.57, 2.41 | <0.001 | 1.54 | 1.23, 1.93 | <0.001 |
| *Place of delivery* |  |  |  |  |  |  |
| At home | Ref. |  |  |  |  |  |
| With health facility | 2.11 | 1.69, 2.63 | <0.001 | 1.66 | 1.31, 2.11 | <0.001 |
| *Delivery method* |  |  |  |  |  |  |
| Vaginal | Ref. |  |  |  |  |  |
| C-section | 1.80 | 1.45, 2.23 | <0.001 | 1.39 | 1.10, 1.75 | 0.005 |
| *Postnatal visit after delivery* |  |  |  |  |  |  |
| After 48 hours | Ref. |  |  |  |  |  |
| Within 48 hours | 0.48 | 0.37, 0.62 | <0.001 | 0.66 | 0.49, 0.89 | 0.007 |
| *Postnatal checkup provider* |  |  |  |  |  |  |
| Unprofessional personnel | Ref. |  |  |  |  |  |
| Physicians/ professionals | 2.19 | 1.67, 2.88 | <0.001 | 1.69 | 1.26, 2.26 | <0.001 |

**Note:** Ref.: Reference category; Adjusted by maternal age, occupation and wealth index

**Supplementary Table 9:** Association between maternal care parameters of mothers in time of pregnancy and delivery with ISSSF

| **Characteristics** | **Introduction of solid, semi-solid and soft foods** | | | | | |
| --- | --- | --- | --- | --- | --- | --- |
|  | **Crude** | | | **Adjusted** | | |
|  | OR | 95% CI | *p*-value | OR | 95% CI | *p*-value |
| *Number of antenatal visits* |  |  |  |  |  |  |
| 0-3 | Ref. |  |  |  |  |  |
| ≥4 | 1.24 | 1.05, 1.46 | 0.011 | 1.24 | 1.04, 1.48 | 0.016 |
| *Place of delivery* |  |  |  |  |  |  |
| At home | Ref. |  |  |  |  |  |
| With health facility | 1.09 | 0.92, 1.28 | 0.304 | 1.12 | 0.93, 1.34 | 0.203 |
| *Delivery method* |  |  |  |  |  |  |
| Vaginal | Ref. |  |  |  |  |  |
| C-section | 1.18 | 0.99, 1.40 | 0.066 | 1.22 | 1.02, 1.48 | 0.034 |
| *Postnatal visit after delivery* |  |  |  |  |  |  |
| After 48 hours | Ref. |  |  |  |  |  |
| Within 48 hours | 0.79 | 0.63, 1.00 | 0.052 | 0.76 | 0.59, 0.97 | 0.030 |
| *Postnatal checkup provider* |  |  |  |  |  |  |
| Unprofessional personnel | Ref. |  |  |  |  |  |
| Physicians/ professionals | 1.25 | 1.02, 1.53 | 0.032 | 1.30 | 1.04, 1.62 | 0.019 |

**Note:** Ref.: Reference category; Adjusted by maternal age, occupation and wealth index
